# Clinical and Radiological Outcomes of C1–C2 Fixation: 3D-Printed Template vs. Free-Hand Technique

**DOI:** 10.3390/jcm15020408

**Published:** 2026-01-06

**Authors:** Ceren Kizmazoglu, Koray Ur, Inan Uzunoglu, Bugra Husemoglu, Ersin Ikizoglu, Musa Sezer, Ege Coskun, Mert Arslan, Hatun Mine Sahin, Ercan Ozer

**Affiliations:** 1 Department of Neurosurgery, Dokuz Eylul University School of Medicine, 35340 Izmir, Turkey; urkoray@gmail.com (K.U.); ersin.ikizoglu@gmail.com (E.I.); ege.coskun.92@gmail.com (E.C.); mertarslandr@gmail.com (M.A.); sahinhatunmine@gmail.com (H.M.S.); ercan.ozer@deu.edu.tr (E.O.); 2 Department of Neurosurgery, Ankara Etlik City Hospital, 06170 Ankara, Turkey; dr_inan_uzunoglu@hotmail.com (I.U.); sezer.musa1299@gmail.com (M.S.); 3 Department of Biomechanics, Institute of Health Sciences, Dokuz Eylul University, 35340 Izmir, Turkey; bugrahusem@gmail.com

**Keywords:** 3D model, 3D printing, C1–C2 fixation, patient-specific template

## Abstract

**Objectives:** The Goel–Harms technique provides rapid stabilization and high fusion rates for atlantoaxial instability but carries a risk of neurovascular injury during lateral mass and pedicle screw insertion. Recently, 3D printing has emerged as a cost-effective and increasingly accessible tool in various surgical fields. This study aimed to compare the clinical and radiological outcomes of C1–C2 fixation using a 3D-printed template versus the free-hand technique. **Methods:** This retrospective cohort study included patients who underwent C1–C2 fixation with the Goel–Harms technique at two tertiary neurosurgical centers between 2021 and 2023. Operative, radiological, and functional outcomes were reviewed in 21 patients who were operated using either a patient-specific 3D-printed template applied intraoperatively (Group 1; *n* = 10) or the free-hand technique (Group 2; *n* = 11). Postoperative screw accuracy was assessed using the Gertzbein–Robbins classification. **Results:** A total of 84 screws were placed (Group 1: 40; Group 2: 44). In Group 1, 38 of 40 screws (95%) were accurately placed, compared with 41 of 44 screws (93.1%) in Group 2. The mean fluoroscopy and operative times were significantly shorter in Group 1 than in Group 2 (21.90 ± 4.33 s vs. 27.09 ± 13.48 s, *p* = 0.012; 126.60 ± 28.70 min vs. 171.36 ± 40.44 min, *p* = 0.010, respectively). **Conclusions:** The 3D-printed template technique significantly reduced operative and fluoroscopy times compared with the free-hand technique. Three-dimensional printing offers a cost-effective alternative to conventional navigation systems by eliminating their time-consuming preoperative setup in the operating room.

## 1. Introduction

Craniovertebral junction (CVJ) anomalies typically develop due to congenital deformities, trauma, tumors, infections, or rheumatologic degeneration. Atlantoaxial dislocation and basilar invagination are the most common CVJ anomalies, which may present with benign clinical manifestations like neck pain or restricted neck movements to more severe ones like spastic quadriparesis resulting from cervicomedullary compression, respiratory distress, and even sudden death [1,2]. Surgical interventions in this region demand advanced expertise due to its complex anatomy and the narrow surgical corridor created by the proximity of critical structures, such as the spinal cord, brainstem, and vertebral arteries [3].

In the last 20 years, C1–C2 screw–rod instrumentation has become one of the most widely adopted techniques for the surgical treatment of CVJ anomalies [4,5,6]. However, despite the availability of advanced imaging techniques and surgical methods that have led to improved patient outcomes, surgeries performed with the free-hand technique are prone to significant complications, including the risk of iatrogenic neurovascular injury, postoperative neurological deficits, intraoperative blood loss, and screw malposition, all of which contribute to increased morbidity and mortality [7].

Recently, 3D printing technology has been increasingly adopted across various surgical disciplines [1,2,3,8]. This technique enables the creation of highly precise, patient-specific implants and models to enhance surgical safety and intraoperative anatomical understanding; supports surgical planning; and facilitates patient education regarding the underlying pathology. Evidence regarding the reliability of 3D-printed templates as a cost-effective alternative to conventional navigation systems in C1–C2 fixation remains scarce in the current literature. In this study, we created patient-specific 3D-printed guide templates and applied them intraoperatively in patients undergoing C1–C2 fusion with the C1 lateral mass–C2 pedicle screw fixation technique. We compared the impact of the 3D-printed template technique with the traditional free-hand technique in terms of the clinical and radiological outcomes of C1–C2 instrumentation.

## 2. Materials and Methods

### 2.1. Study Design and Sample

We retrospectively reviewed the clinical and radiological data of 21 patients who underwent C1–C2 fixation using the Goel–Harms method for atlantoaxial pathologies between 2021 and 2023 at two different centers. Because this was a retrospective study, no a priori sample size calculation was performed. All consecutive patients meeting the eligibility criteria during the study period were included, resulting in a total of 21 patients. All surgeries were performed by experienced neurosurgeons. Ethical approval for the study was obtained from the local institutional review board (Approval No: 2024/02-01, approval date 8 February 2024), and informed consent was obtained from all patients. The patients were categorized into two groups based on the surgical technique used: group 1 (*n* = 10), using preoperatively prepared 3D-printed templates; and group 2 (*n* = 11), using the free-hand technique.

### 2.2. Imaging Data

At both centers, all patients underwent a standard imaging protocol of preoperative magnetic resonance imaging and 1 mm slice computed tomography (CT) angiography of the cervical region to visualize the regional bony structures, as well as the course of the vertebral artery, to detect any potential vascular variations or anomalies.

### 2.3. Preparation and Printing of 3D Models

**Figure 1:**
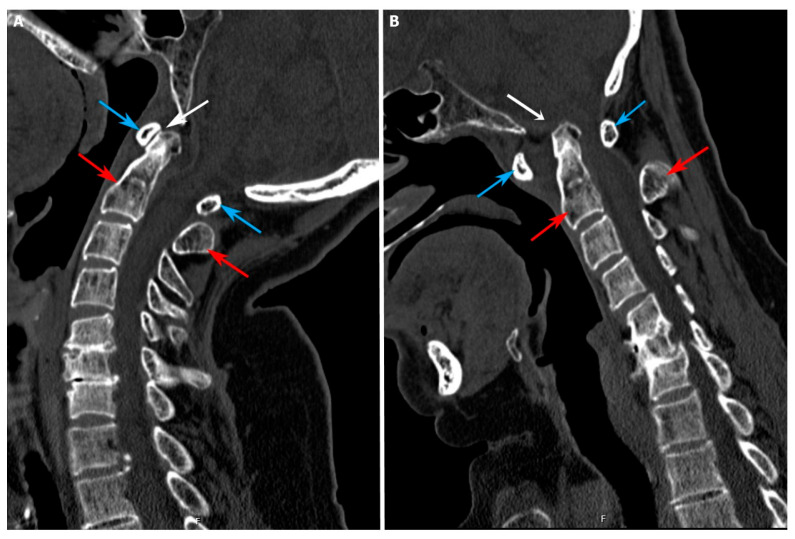
Atlantoaxial dislocation on dynamic computed tomography (CT) imaging. (**A**): Extension view; (**B**): flexion view. White arrow: change in the atlantodental interval (ADI); Blue arrow: C1 vertebra; Red arrow: C2 vertebra.

**Figure 2:**
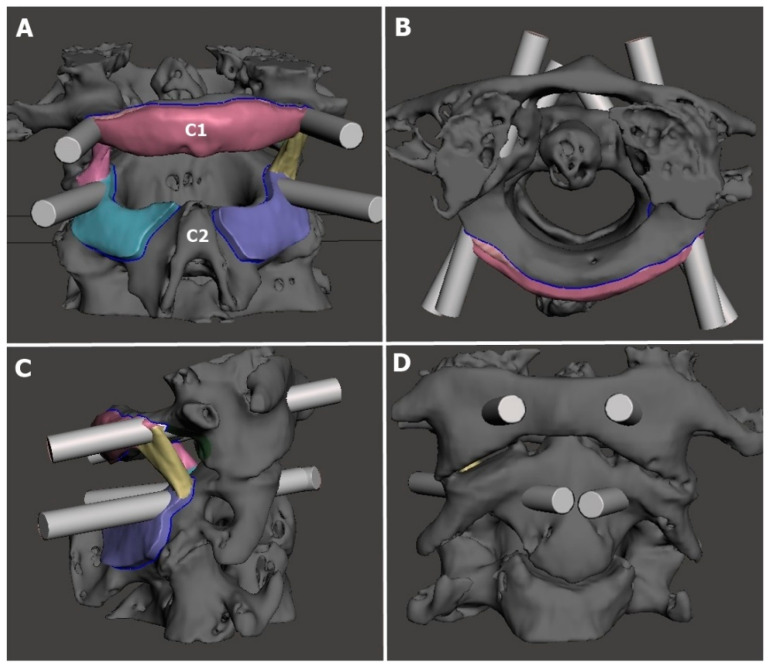
Simulation of screw trajectory guide templates created using the Meshmixer software. (**A**): Posterior view; (**B**): superior view; (**C**): right lateral view; (**D**): anterior view.

**Figure 3:**
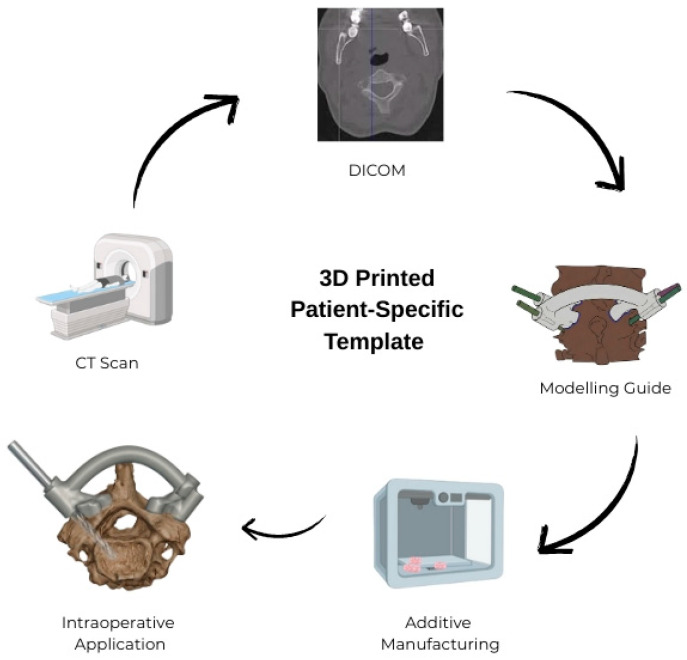
Workflow of the design, manufacturing, and intraoperative application of the 3D-printed templates.

**Figure 4:**
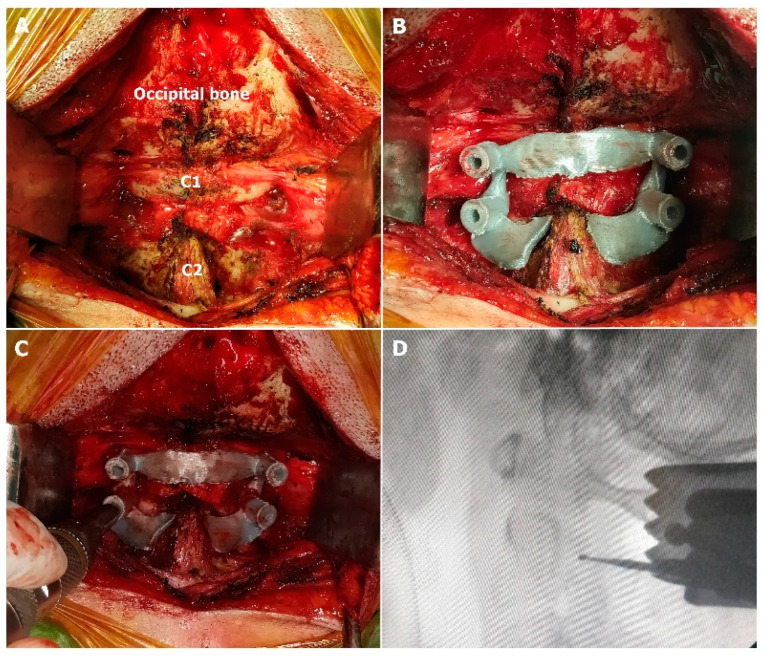
Intraoperative application of the guide template. (**A**): Exposure of the surgical field; (**B**): placement of the 3D-printed template; (**C**): creation of pilot burr holes using a high-speed drill; (**D**): advancement of the high-speed drill to a depth of 12 mm.

**Figure 5:**
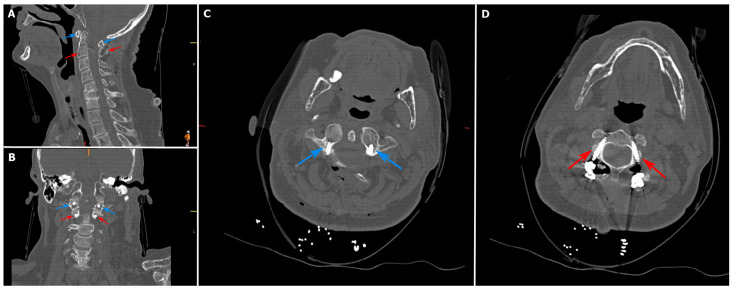
Grade A Gertzbein and Robbins screws applied in the study cohort. (**A**): Corrected atlantodental interval on sagittal computed tomography (CT); (**B**): screw positioning on coronal CT; (**C**): placement of C1 screws; (**D**): placement of C2 screws. Blue arrow: C1 vertebra and screws; Red arrow: C2 vertebra and screws.

The Digital Imaging and Communications in Medicine images obtained from the CT scans were first processed for segmentation using Mimics (version 14; Materialise, Leuven, Belgium) and then subjected to surface optimization. Next, the individualized anatomical models were exported in the Surface Tessellation Language format using MeshMixer (v3.5, Autodesk, San Francisco, CA, USA), followed by the creation of patient-specific surgical guide templates. The digital models of the 3D guide templates were then imported into Ultimaker Cura (version 5.0; Utrecht, The Netherlands) to generate a G-code file for 3D printing. The G-code files were transferred to an Ultimaker 2 3D printer (Ultimaker B.V., Utrecht, The Netherlands) based on Fused Deposition Modeling (FDM) technology; the physical models were printed using polylactic acid thermoplastic filament with a layer height of 0.1 mm and an infill density of 80% (Figure 1, Figure 2 and Figure 3; Figure 1, Figure 2, Figure 4 and Figure 5 are images of the same patient). 3D-printed templates were then sterilized with ethylene oxide and prepared for intraoperative use. Template design and production were completed in one day, and printing and sterilization took an additional day. The ready-to-use template was delivered to the surgical team within 48 h at a cost of approximately USD 90.

### 2.4. Data Collection

For all patients, we collected data regarding general demographic characteristics, underlying causes of atlantoaxial instability, any predisposing conditions, operative time, intraoperative blood loss, complications, and fluoroscopy time. Additionally, CT imaging was used to assess the C1 posterior arch height, minimum width of the C1 lateral mass, minimum width of the C2 pedicle, atlantodental interval (ADI), and cervicomedullary angle.

ADI (horizontal dislocation; normal: <3 mm) and cervicomedullary angle (to evaluate spinal cord and medulla compression due to dens dislocation; normal: >140°) were measured using sagittal CT scans; both preoperative and postoperative values were recorded for all patients.

The course of the vertebral artery was assessed to identify patients with a high-riding vertebral artery; this condition was defined as an isthmus height of <5 mm and/or an internal C2 height of <2 mm in sagittal CT sections passing 3 mm lateral to the lateral border of the spinal canal [9].

Postoperative functional outcomes were assessed using the Visual Analog Scale Score for Neck Pain (VASSNP), the Nurick scale, neck stiffness, and the Japanese Orthopaedic Association (JOA) scores (preoperative and postoperative).

### 2.5. Surgical Technique

In group 1, the surgical procedure involved the following sequence (Figure 4 and Figure 5):

Step 1: Exposing the surgical field using monopolar cautery.

Step 2: Placing the 3D-printed template as a guide onto the C1 posterior arch and the C2 lamina.

Step 3: Creating pilot burr holes using a high-speed (2 mm round aggressive) drill, followed by fluoroscopic verification while the drill was still in place.

Step 4: Advancing the high-speed drill (2 mm round diamond) to a depth of 12 mm.

Step 5: Manually inserting a 2 mm bone tap to the predetermined screw length. (Following steps 3, 4, and 5, the bone was probed with a feeler to verify its integrity.).

Step 6: Removing the 3D-printed templates and inserting screws according to the preoperatively determined lengths via the established pilot burr holes.

In group 2, the surgery was performed by an experienced surgeon using the free-hand technique under intraoperative fluoroscopic guidance [4,10,11].

Postoperative CT scans were obtained for all patients, and the accuracy of the screw placement was evaluated using Gertzbein and Robbins’ classification scale, which was assessed in a blinded manner to avoid bias [12].

- Grade A: Completely intrapedicular position without cortical breach.
- Grade B: Cortical breach < 2 mm.
- Grade C: Cortical breach between 2 and 4 mm.
- Grade D: Cortical breach between 4 and 6 mm.
- Grade E: Cortical breach > 6 mm or screw located entirely outside the pedicle.

Screws classified as Grade A or B were regarded as accurately placed, while Grades C, D, and E were categorized as misplaced. Screws that contacted the vertebral artery or caused spinal canal compression were also noted.

### 2.6. Statistical Analysis

Statistical analyses were performed using the Statistical Package for the Social Sciences (version 29.0; IBM Corp., Chicago, IL, USA). Mean values and standard deviations (SDs) were calculated for all data, as the data were normally distributed. Differences between preoperative and postoperative ADI values were assessed using the Wilcoxon signed-rank test, whereas between-group differences were analyzed using the Mann–Whitney U test. A *p*-value of <0.05 was considered statistically significant.

## 3. Results

The mean age of the 21 patients was 54.57 ± 18.69 years; among these patients, there were 10 (47.6%) females and 11 (52.4%) males. Eight patients (38.1%) had atlantoaxial dislocation, ten (47.6%) had fractures, two (9.5%) had cervical tumors, and one (4.8%) had atlas assimilation (Table 1).

Table 2 presents a comparison of the pre- and postoperative radiological and intraoperative variables between the two groups.

A total of 84 screws were placed in the 21 patients; the postoperative classification of all screws was performed according to the Gertzbein and Robbins scale. In group 1, a total of 20 C1 and 20 C2 screws were placed using the 3D-printed template; all 20 C1 screws (100%) were classified as Grade A, whereas for C2 screws, 18 (90%) were deemed as Grade A, 1 (5%) as Grade C, and 1 (5%) as Grade D. In group 2 (*n* = 11), 22 C1 and 22 C2 screws were placed using the free-hand technique. For C1 screws, 19 screws (86.4%) were deemed as Grade A, 2 (9.1%) as Grade B, and 1 (4.5%) as Grade C; regarding C2 screws, 17 (77.3%) were deemed as Grade A, 3 (13.6%) as Grade B, 1 (4.5%) as Grade D, and 1 (4.5%) as Grade E (Table 3).

**Figure 6:**
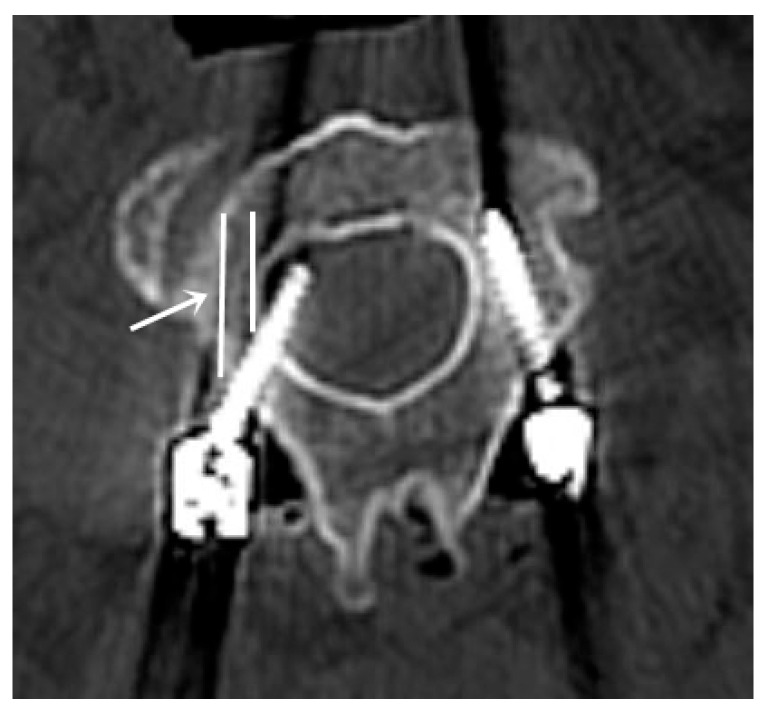
In one patient in the 3D-printed template group, a screw could not be inserted into the trajectory prepared with the pilot burr hole. The white arrow indicates the trajectory prepared using the 3D-printed template.

A total of five screws were identified as misplaced across both groups. In group 1, in one patient, the C2 screw trajectory deviated medially from the preplanned path, despite pilot burr hole preparation, and postoperative CT confirmed it as a misplaced screw (Grade D; Figure 6). Since the patient had a wide spinal canal and no clinical symptoms, no revision was performed. In group 2, one C1 screw penetrated the atlanto-occipital joint, leading to postoperative pain. Additionally, two C2 screws (Grade D and E) in group 2 were revised postoperatively due to postoperative pain and neurological deficits; their positioning was re-evaluated postoperatively.

In group 2, two patients with misplaced screws experienced neck pain and arm numbness, which resolved with steroid treatment; however, no neurological deficits were observed in any patient. No vertebral artery injuries occurred in any case; however, one patient suffered significant intraoperative bleeding from the vertebral venous plexus and developed a cerebral infarction in the first postoperative week. No evidence of vasospasm in the vertebral artery was detected, and no endovascular intervention was required.

Regarding postoperative functional outcomes, no significant differences were observed in VASSNP, Nurick scale, or neck stiffness scores (Table 4). In terms of group characteristics, the only significant difference between the groups was found in preoperative and postoperative JOA scores (*p* = 0.006 and *p* = 0.025, respectively); however, these differences reflect baseline group characteristics rather than the outcomes of the surgical techniques compared in this study.

The mean intraoperative blood loss was lower in group 1 than in group 2, although the difference was not statistically significant (*p* = 0.192). Furthermore, the mean fluoroscopy time (21.90 ± 4.33 s versus 27.09 ± 13.48 s; *p* = 0.012) and the mean operative time (126.60 ± 28.70 min versus 171.36 ± 40.44 min; *p* = 0.010) were significantly shorter in group 1 than in group 2.

## 4. Discussion

Posterior C1–C2 fixation is considered the gold standard for treating atlantoaxial instability. In clinical practice, the Goel–Harms technique—involving C1 lateral mass and C2 pedicle screw fixation—is widely used because of its high fusion rates [4,10]. Magerl and Seeman first described the transarticular screw-fixation technique for C1–C2 fusion [13]; it allows screw placement without opening the venous plexus, thereby minimizing intraoperative bleeding. However, the risk of vertebral artery injury (1.3–8.2%) has led to the adoption of alternative techniques [14].

Pan et al. [11] modified the entry point of the C1 lateral mass screw to the notch of the C1 posterior arch to minimize venous plexus bleeding and reduce C2 nerve root irritation. This modification has been associated with a lower risk of vertebral artery injury and facilitates reduction maneuvers. It is particularly relevant during C2 screw placement, where the risk of vertebral artery injury is higher—especially in the presence of a high-riding vertebral artery—necessitating careful selection of the screw entry point.

Akinduro et al. [15] conducted a meta-analysis of 27 studies comprising 2078 cases and reported vertebral artery injury in 60 patients (2.9%). Among them, 10% experienced ipsilateral stroke, 6.7% had transient motor weakness, and 8.3% developed an arteriovenous fistula or pseudoaneurysm. Endovascular intervention was required in 13.3% of these cases, and mortality occurred in four patients (6.7%). Other studies have reported vertebral artery injury rates of 2% with the Goel–Harms technique and 4.1% with transarticular screw fixation [16].

Three-dimensional (3D) printed models enable preoperative visualization of anatomical variations and allow surgeons to interact with tangible, patient-specific structures. Custom drill guide templates ensure precise screw placement and help minimize complications. Because these templates are designed based on the 3D anatomy of the lamina, they are unaffected by flexion or extension and remain stable against torsional forces during high-speed drilling. In our technique, screws were inserted directly into the holes created after drilling; therefore, no separate screw guide template was printed. In one patient, this led to a slightly more medial trajectory of the C2 screw than planned; however, no vascular injury occurred. In two patients, C2 screws placed using the free-hand technique required revision due to postoperative pain and neurological deficits. No ischemic events or vascular complications were observed during either early or long-term follow-up in patients treated with 3D-printed templates.

According to the literature, the Mazor robotic navigation system allowed for a pedicle screw placement accuracy rate of 97–99%, as graded by the Gertzbein and Robbins classification [17,18]. However, this system has two notable limitations. The first is “shift,” which refers to a change in the relative position between the Mazor X Stealth Edition (MXSE) system and the patient; this can occur at various stages of the procedure, often due to accidental contact or leaning on the system. Maintaining a stable physical connection between the patient and the system can help mitigate this issue. The second common error is “skiving,” which occurs when instruments such as the cannula, drill, tap, or guide deviate from the intended trajectory due to downward forces applied at the bone interface; this can result from translational forces exerted on the tools by the surgeon or surrounding soft tissues.

Keric et al. [19] performed 413 surgeries using robotic assistance and reported the average operative time as 258.7 ± 105.6 min, and the mean fluoroscopy time as 114.4 ± 72.5 s. The authors also noted that robot registration failure was more frequent in patients with a body mass index of >35–40 kg/m^2^.

Pai et al. [20] reported the placement of 206 cervical screws with robotic assistance; the mean total surgical time was noted as 190 min (range: 150–240 min), with 40 min (range: 30–55 min) required for cervical exposure. The average time per screw was 6 min (range: 5–8 min); the time required from robotic arm setup to screw planning was 25 min (range: 20–30 min); and the time for O-arm positioning and imaging was 10 min (range: 8–15 min). Decompression procedures took an average of 45 min (range: 35–60 min). The mean intraoperative blood loss was 180 mL (range: 120–300 mL).

In our study, although the blood loss in the 3D-printed template group was lower than in the free-hand group, the difference was not statistically significant (*p* = 0.192). However, the mean fluoroscopy time was significantly shorter in the 3D-printed template group (21.90 ± 4.33 s) compared to the free-hand group (27.09 ± 13.48 s; *p* = 0.012).

Garufi et al. [21] compared the outcomes of robot-assisted and O-arm neuronavigation-guided lumbar fusion surgeries. The total screw placement time was significantly shorter with neuronavigation compared to robotic assistance (37.42 min versus 57.23 min; *p* < 0.01). However, the average time per screw (2.90 vs. 3.25 min, *p* = 0.37) did not differ significantly between the two methods. In our study, the mean operative time was significantly shorter in the 3D-printed template group than in the free-hand group (126.60 ± 28.70 min versus 171.36 ± 40.44 min; *p* = 0.010).

Another study evaluated 5132 pedicle screws placed with O-arm navigation or the free-hand technique [22]. They reported the accuracy rate as 95.2% in the navigation group and 86.9% in the free-hand group. The main cause of misplacement in the intraoperative CT group was the torsional distortion of spinal alignment during drilling, tapping, or screw insertion.

Previous studies have reported pedicle screw breach rates of 3.2–9% using intraoperative CT [23,24]. Sugawara et al. developed a technique using location, drill, and screw guide templates for C1–C2 fixation, achieving highly accurate placement with a mean deviation of only 0.7 mm [25]. The pedicle breach rate for this technique was 1.3%; however, template production with this method takes approximately 2–3 days, including 1–2 days for design and an additional day for 3D printing. In our study, template designing and production were completed within one day at the biomechanical laboratory, followed by an additional day for printing and sterilization. Thus, a ready-to-use template was delivered to the surgical team within 48 h at a cost of approximately USD 90.

Buchmann et al. [26] reported an accuracy rate of 94.2% (Gertzbein and Robbins classification Grades A and B) in 127 patients who underwent free-hand C1–C2 fixation. In the literature, navigation-assisted techniques show accuracy rates ranging from 90.5% to 100% [27,28,29]. Buchmann et al. also documented a mean operative time of 100 min, whereas the average operative times for navigation-assisted surgeries have been reported as approximately 130–306 min [26,29,30,31].

Several engineering software programs have been used to create 3D navigation templates, with most programs typically requiring manual adjustments for determining screw trajectories. Previous studies have reported accuracy rates between 84% and 97.6% [32,33,34,35]. In our study, the accuracy rate was 95% in the 3D-printed template group compared to 93.1% in the free-hand group, based on Gertzbein and Robbins classification Grades A and B; however, this difference was not statistically significant (*p* = 0.209).

In group 1, the right C2 screw in one patient deviated from the intended trajectory designed by the guide. This deviation was attributed to a limitation in our template method: the guide was designed only for pilot-hole drilling, and no additional screw trajectory guide was produced. In some patients, osteoporosis caused segmentation difficulties during software-based planning, which prolonged preoperative preparation for guide generation. The time taken to deliver the ready-to-use template to the surgical team is approximately 48 h, which represents a limitation for its application in emergency cases. Because of the small sample size, a formal power analysis was not feasible. This study was based solely on retrospectively evaluated operated cases; therefore, it should be considered a pilot investigation with inherent limitations related to its retrospective design and small cohort, which also restrict the strength of safety-related conclusions.

## 5. Conclusions

In this study, the use of 3D-printed templates for C1–C2 fixation resulted in reduced fluoroscopy time and, consequently, operative time. These templates offer a significant advantage in minimizing complications, particularly for less experienced surgeons, while also enhancing procedural efficiency for experienced surgeons. Furthermore, 3D printing systems are more cost-effective than traditional navigation systems and do not require intraoperative preparation, offering a practical and efficient alternative to navigation-assisted techniques. Future randomized, multicenter studies with larger cohorts are warranted to validate the present findings and to further define the clinical role of 3D-printed templates in C1–C2 fixation.

## Figures and Tables

**Table 1:** Baseline demographic and clinical characteristics of the study patients (N = 21).

Characteristic	3D-Printed Template (*n* = 10)	Free-Hand (*n* = 11)	*p*-Value
Age (years), mean ± SD	47.70 ± 20.81	60.81 ± 14.79	0.173
Sex, *n* (%)			0.835
Male	5 (50.0%)	6 (54.5%)	
Female	5 (50.0%)	5 (45.5%)	
Etiology, *n* (%)			0.716
Atlantoaxial dislocation	4 (40.0%)	4 (36.4%)	
Fracture	4 (40.0%)	6 (54.5%)	
Tumor	1 (10.0%)	1 (9.1%)	
Atlas assimilation	1 (10.0%)	0 (0.0%)	
Comorbidity, *n* (%)			0.835
Yes	5 (50.0%)	5 (45.5%)	
Presenting symptoms, *n* (%)			0.784
Occiput/neck pain	5 (50.0%)	7 (63.6%)	
Extremity weakness	3 (30.0%)	2 (18.2%)	
Paresthesia	2 (20.0%)	2 (18.2%)	

**Table 2:** Comparison of the pre- and postoperative radiological and intraoperative parameters between the study groups (N = 21).

Parameter (Mean ± SD)	3D-Printed Template (*n* = 10)	Free-Hand (*n* = 11)	*p*-Value
C1 posterior arch height (mm)	7.31 ± 1.98	7.34 ± 1.28	0.944
Minimum C1 lateral mass width (mm)	7.59 ± 1.55	7.16 ± 1.03	0.341
Minimum C2 pedicle width (mm)	4.34 ± 0.86	4.48 ± 0.50	0.502
Preoperative atlantodental interval (mm)	6.01 ± 3.98	2.64 ± 1.66	0.062
Postoperative atlantodental interval (mm)	2.37 ± 2.24	1.90 ± 1.10	0.621
Preoperative cervicomedullary angle (°)	138.00 ± 11.50	143.09 ± 8.61	0.230
Postoperative cervicomedullary angle (°)	149.70 ± 17.95	144.90 ± 10.51	0.324
Operative time (min)	126.60 ± 28.70	171.36 ± 40.44	0.010
Intraoperative blood loss (mL)	324.54 ± 152.99	412.50 ± 97.53	0.192
Fluoroscopy time (s)	21.90 ± 4.33	27.09 ± 13.48	0.012

**Table 3:** Accuracy of screw placement per vertebral level according to the Gertzbein–Robbins classification (N = 84).

Level	Number of Screws (*n*)	Grade A, *n* (%)	Grade B, *n* (%)	Grade C, *n* (%)	Grade D, *n* (%)	Grade E, *n* (%)
3D-printed template—C1 screw	20	20 (100%)	0 (0%)	0 (0%)	0 (0%)	0 (0%)
3D-printed template—C2 screw	20	18 (90.0%)	0 (0%)	1 (5.0%)	1 (5.0%)	0 (0%)
Free-hand—C1 screw	22	19 (86.4%)	2 (9.1%)	1 (4.5%)	0 (0%)	0 (0%)
Free-hand—C2 screw	22	17 (77.3%)	3 (13.6%)	0 (0%)	1 (4.5%)	1 (4.5%)

**Table 4:** Comparison of functional outcomes between the study groups (N = 21) (VASSNP: Visual Analog Scale Score for Neck Pain; JOA: Japanese Orthopaedic Association Score; Preop: preoperative; Postop: postoperative; SD: standard deviation).

Variable	3D-Printed Template (*n* = 10)	Free-Hand (*n* = 11)	*p*-Value
VASSNP, mean ± SD	2.30 ± 1.33	2.36 ± 1.20	0.942
Nurick Scale, *n* (%)			0.806
Grade 0	6 (60.0%)	6 (54.5%)	
Grade 1	4 (40.0%)	5 (45.5%)	
Neck Stiffness, *n* (%)			0.741
None	6 (60.0%)	7 (63.6%)	
Mild	3 (30.0%)	4 (36.4%)	
Severe	1 (10.0%)	0 (0.0%)	
Preop JOA Score, mean ± SD	13.40 ± 5.10	17.45 ± 0.68	0.006
Postop JOA Score, mean ± SD	14.80 ± 4.44	17.90 ± 0.30	0.025

## Data Availability

The data supporting the findings of this study are available from the corresponding author upon reasonable request.

## Abbreviations

CVJ	Craniovertebral junction
CT	Computed tomography
ADI	Atlantodental interval
VASSNP	Visual Analog Scale Score for Neck Pain
JOA	Japanese Orthopaedic Association
SD	Standard deviations
